# Determination of the Heterogeneity of Intramuscular Fat and Visceral Adipose Tissue From Dezhou Donkey by Lipidomics and Transcriptomics Profiling

**DOI:** 10.3389/fnut.2021.746684

**Published:** 2021-09-28

**Authors:** Mengmeng Li, Mingxia Zhu, Wenqiong Chai, Yonghui Wang, Yinghua Song, Baoxiu Liu, Changyun Cai, Yingzi Song, Xue Sun, Peng Xue, Changfa Wang

**Affiliations:** Liaocheng Research Institute of Donkey High-Efficiency Breeding and Ecological Feeding, College of Agronomy, Liaocheng University, Liaocheng, China

**Keywords:** Dezhou Donkey, lipidomics, transcriptomics, heterogeneity, intramuscular fat, visceral adipose tissue

## Abstract

Intramuscular fat (IMF) and visceral adipose tissue (VAT) are both lipids, but have significantly different deposition processes. Furthermore, the heterogeneity of lipid molecular characteristics and mechanisms is unclear. Accordingly, this study used non-targeted lipidomics and transcriptomics to analyze the lipid profiles and metabolism of longissimus dorsi muscle (LDM) and VAT from donkeys. A total of 1,146 and 1,134 lipids belonging to 18 subclasses were identified in LDM and VAT, respectively, with LDM having higher glycerophospholipid (GP) and lower glycerolipid (GL) contents. Polyunsaturated fatty acids (PUFAs) were distributed preferentially at the sn-1 positions in triglycerides (TGs), and sn-2 positions in phosphatidylcholine (PC) and phosphatidylethanolamine (PE). The percentage PUFA content in TGs was significantly lower in LDM than in VAT, while the opposite trend was observed for PUFAs in PC and PE. A total of 110 different lipid molecules (72 downregulated and 38 upregulated) were identified in LDM compared with VAT, of which 11 were considered potential lipid markers. These different lipids were involved in 17 metabolic pathways, including GL and GP metabolisms. Of the 578 differentially expressed genes screened, 311 were downregulated and 267 were upregulated in LDM compared with VAT. Enriched ontology analysis of the differentially expressed genes mainly involved sphingolipid signaling pathways, and GP, GL, and sphingolipid metabolisms. Overall, lipidomics and transcriptomics indicated differences in lipid profiles and metabolism in LDM and VAT, providing new perspectives for the study of heterogeneity in IMF and VAT.

## Introduction

Meat products are important sources of fat in the human diet globally, with the fat content playing a key role in the overall palatability of meat. Intramuscular fat (IMF) positively affects the quality and nutritional value of meat, including juiciness, flavor, tenderness, and fatty acid profiles (1, 2). IMF is mainly composed of triglycerides (TGs) and phospholipids, and is rich in phospholipids, palmitic acid, stearic acid, oleic acid, and polyunsaturated fatty acids (PUFA) compared with other adipose tissues, such as visceral adipose tissue (VAT) (3, 4). The differences in IMF and VAT deposition reflect the heterogeneity of the lipidome, especially lipid molecules including glycerophospholipids (GPs) and sphingolipids (SLs), which is unclear.

Recently, to improve meat quality, improving the IMF content of livestock and poultry, and determining the internal molecular mechanisms involved, have become hot topics in genetics, nutrition, and other fields. Previous studies have suggested that the IMF content varies widely depending on animal breed or genetics, slaughter age and weight, nutrition levels, and other factors (5–7). Some candidate genes for IMF deposition have been identified, such as heart and adipocyte fatty acid binding proteins (H-FABP and A-FABP, respectively), fatty acid synthase (FAS), hormone sensitive lipase (HSL), lipoprotein lipase (LPL), and peroxisome proliferator-activated receptor (PPAR) (8). Lipid deposition is a process essentially comprising pathways for the synthesis of TGs and GPs from esterified fatty acids of the glycerol skeleton, which are catalyzed by a series of enzymes, including glycerol-3-phospholipid transferase (GPAT), acylglycerol-3-phosphate transferase (AGPAT), and diacylglyceryl transferase (DGAT) in the TG pathway, and choline phosphate transferase (CHPT), ethanolaminephosphate transferase (EPT), and lysophosphatidylcholine transferase (LPCAT) in the GP pathway (9). DGAT is the rate-limiting enzyme in TG synthesis and has been identified as a candidate gene for IMF deposition (10). Furthermore, AGPAT has been reported to play a key role in fatty acid deposition and is a key regulatory factor of lipid metabolism in muscle (11). These findings indicate that genes involved in TG and GP metabolisms in muscle play important roles in regulating IMF deposition.

In recent decades, lipidomics has been successfully applied in various fields to analyze exquisite changes in lipids at the molecular level, especially in food and nutrition science (12). Many studies have used lipidomics to clarify lipid profile changes in donkey milk during lactation (13) and egg yolk in different diets (14), and differentiate domestic pork (15), and chicken (16). A recent study reported the transcriptome atlas of 16 Dezhou Donkey tissues (17). However, few studies have used lipidomics to analyze differences between IMF and VAT. Therefore, the present study used liquid chromatography–mass spectrometry (LC-MS)-based lipidomics detection of IMF and VAT from donkey to analyze differences in the amounts, classes, fatty acid distributions, and metabolism pathways of lipids. Furthermore, transcriptomics analysis was used to analyze differentially expressed genes and their functional enrichment in IMF and VAT. Subsequently, IMF and VAT were distinguished at the lipid molecular level and key regulating pathways were determined by combining lipidomics and transcriptomics. This study investigated the heterogeneity of IMF and VAT at the molecular level, and provides a new perspective for understanding and improving IMF content.

## Materials and Methods

### Animal and Sample Collection

All experimental procedures involving donkeys were approved by the Liaocheng University Animal Care and Use Committee. Thirty-six male Dezhou donkeys, ~18 months old and of similar weight (150 ± 20 kg), were randomly divided into three groups of 12 donkeys each. All donkeys were fed a diet of 70% silage corn straw and 30% corn flour, and were free to eat and drink under the same living conditions. The experiment was conducted at a local farm in Liaocheng City, Shandong Province, China for 6 months.

After the experiment, all donkeys were left unfed for 12 h and weighed (230 ± 31 kg). Three donkeys from each group were randomly selected and transported to Shandong Dong'e Tianlong Food Co., Ltd. After slaughter, LDM and VAT samples were collected, immediately snap frozen in liquid nitrogen, and stored at −80°C for lipidomic and transcriptomic analysis.

### Lipid Extraction and lipidomic Assay

Muscle samples were ground with chloroform/methanol (2:1, *v/v*) using a Xinzhi high-flux tissue grinder (Ningbo, China) at 4°C. Lipids were extracted on ice for 3 h, centrifuged at 8,000 ×g for 20 min at 4°C, and the resultant supernatant was concentrated to dryness under vacuum. The lipids were then dissolved in isopropanol (200 μL) and stored at −80°C for subsequent analysis.

LC-MS was performed according to published methods (18). LC-MS analysis was performed on a AB SCIEX TripleTOF 6600 plus MS (AB SCIEX Inc., Massachusetts, USA) coupled to a UHPLC Nexera Agilent 1290 (Agilent Technologies, Palo Alto, CA, USA) equipped with a Phenomenex Kinetex C18 column (1.7 μm, 100 × 2.1 mm, Agilent). The lipid samples were redissolved in 90% isopropanol/acetonitrile (200 μL) and filtered through a 0.22-μm polyvinylidene fluoride membrane, with 2 and 62 μL of each sample injected for analysis in positive and negative modes, respectively. The autosampler and column temperatures were maintained at 8 and 55°C, respectively. Solvents A and B were acetonitrile/water (60:40, *v/v*) and isopropanol/acetonitrile (90:10, *v/v*), respectively, each containing 0.1% formic acid and 10 mM ammonium formate. Gradient elution was performed using the following mobile phase compositions: 40% solvent B at a flow rate of 0.3 mL/min at 0–1.5 min; increased to 85% solvent B at 1.5–10.5 min; equilibration with 85% solvent B at 10.5–14 min; increased to 100% solvent B at 14–14.1 min; equilibration with 100% solvent B at 14.1–15 min; decreased to 40% solvent B at 15–15.2 min; and finally equilibration with 40% solvent B at 15.2–18 min. To avoid instrumentation error, quality control (QC) samples were prepared by mixing all samples. QC samples were inserted into the detection queue to monitor and evaluate stability and reliability during the experiment.

After LC separation, an AB SCIEX TripleTOF 6600 plus MS was used to measure mass-to-charge ratios (*m/z*). The atomization gas, auxiliary gas, and air curtain gas pressures were 60, 60, and 30 psi, respectively, the temperature was 600°C, and the positive mode, negative mode, and declustering potentials of the ion source were 5, −4.5, and 0.1 kV, respectively. Data acquisition was performed by tandem mass spectrometry (MS/MS), involving a rapid time-of-flight (TOF) MS survey scan. For the TOF MS survey scan, the scanning ranges of positive and negative ion modes were *m/z* 200–2,000 and 100–2,000, respectively. After each scan, 10 fragment patterns (MS2 scan, HCD) were collected to obtain the *m/z* ratios of lipid molecules to lipid fragments. Dynamic background subtraction was applied and the dynamic exclusion method was used to remove noise in MS/MS spectra. Raw data were annotated based on the Lipid Structure Database (LMSD; http://www.lipidmaps.org/) and assessed by orthogonal partial least squares discriminant analysis (OPLS-DA). Lipid species with a variable importance in projection (VIP) of > 1 and *p*-value of <0.05 were identified as statistically significant. The Kyoto Encyclopedia of Genes and Genomes (KEGG) pathway database (https://www.kegg.jp/kegg/) was used to search for enriched metabolites and pathways related to differential lipid molecules.

### RNA-seq Analysis

RNA-seq analysis was performed as previously reported (19). Total RNA extraction and RNA-seq analysis were performed by Shanghai Majorbio Bio-pharm Technology Co., Ltd (Shanghai, China). Briefly, total RNA was extracted from the samples using Trizol reagent (Takara, China). Subsequently, double-stranded cDNA was synthesized by a specific kit (Invitrogen, CA) with random hexamer primers (Illumina). The as-synthesized cDNA was subjected to end-repair, phosphorylation, and “A” base addition sequentially. The cDNA target fragments of 200–300 bp were selected on 2% low range ultra agarose with subsequent PCR amplification. RNA-seq libraries were sequenced in a single lane using an Illumina Novaseq 6000 sequencer (Illumina, San Diego, CA). High-quality sequences (clean reads) were obtained by removing low-quality sequences and connector contamination from the raw reads sequenced using SeqPrep software (https://github.com/jstjohn/SeqPrep). The clean reads were *de novo* assembled using Trinity software (https://github.com/trinityrnaseq/trinityrnaseq) without a reference genome. To annotate the transcriptome, six databases, including the National Center for Biotechnology Information NR database (ftp://ftp.ncbi.nlm.nih.gov/blast/db/), Swiss-Prot protein database (http://web.expasy.org/docs/swiss-prot_guideline.html), Pfam database (http://pfam.xfam.org/), Clusters of Orthologous Groups (COG) of proteins database (http://www.ncbi.nlm.nih.gov/COG/), Gene Ontology (GO) database (http://www.geneontology.org), and KEGG database were searched using BLAST with a cut-off e-value of 10^−5^. Gene expression was given in fragments per kilobase per million mapped fragments (FPKM) using DESeq2. Genes expressed differentially in LDM and VAT were screened using DESeq2. Genes with *p* < 0.05 and |log2(foldchange)| > 1.5 were identified as statistically significant.

### Statistical Analysis

Data were presented as means ± standard error of the mean (SEM), and analyzed by one-way analysis of variance (ANOVA), followed by Tukey's test for pairwise comparisons using SPSS 26.0 software (SPSS Inc., Chicago, IL, USA). *p* < 0.05 was considered statistically significant.

## Results

### Lipid Profiles

**Figure 1:**
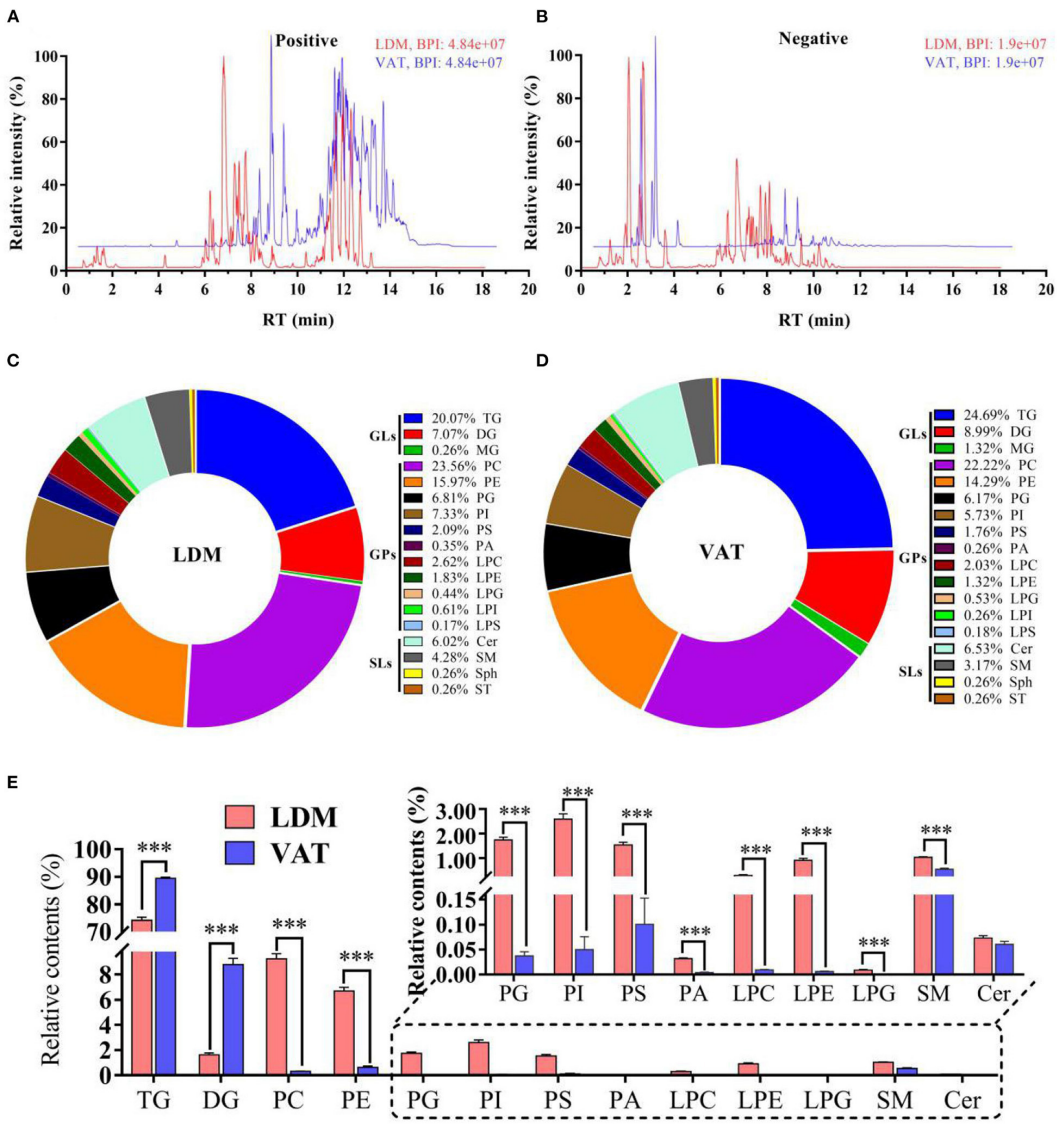
Overall lipid composition and content in LDM and VAT of Dezhou donkey. **(A,B)** Representative base peak diagrams of electrospray ionization in **(A)** positive and **(B)** negative modes. **(C,D)** Percentage of lipid subclasses in **(C)** LDM and **(D)** VAT. **(E)** Relative lipid content (% total lipids) in LDM and VAT of Dezhou donkey. Values are presented as means ± SEM (*n* = 9), ***p < 0.001.

As shown in Figures 1A,B, qualitative lipid analysis achieved excellent separation for IMF and VAT. The OPLS-DA scores were *R*^2^*X* = 0.863, *R*^2^*Y* = 0.993, and *Q*^2^ = 0.892 in positive and negative ion modes, and corresponding OPLS-DA validation plots were applied, providing *R*^2^ and *Q*^2^ intercept parameters of (0.0, 0.63) and (0.0, −0.11), respectively (Supplementary Figures S1A,B). A total of 1,146 and 1,134 lipids, belonging to 18 subclasses, were identified in donkey LDM and VAT, respectively, mainly comprising glycerolipids (GLs), GPs, and SLs (Figures 1C,D). The relative contents of TGs and diacylglycerol (DG) in LDM were significantly lower than those in VAT (*p* < 0.001), while the contents of phosphatidylcholine (PC), phosphatidylethanolamine (PE), phosphatidylglycerol (PG), phosphatidylinositol (PI), phosphatidylserine (PS), phosphatidic acid (PA), lysophosphatidylcholine (LPC), lysophosphatidylethanolamine (LPE), lysophosphatidylglycerol (LPG), and sphingomyelin (SM) in LDM were significantly higher than those in VAT (*p* < 0.001; Figure 1E). The relative ceramide (Cer) contents showed no significant difference between the two groups (*p* > 0.05).

### Positional Distribution of Fatty Acids

**Figure 2:**
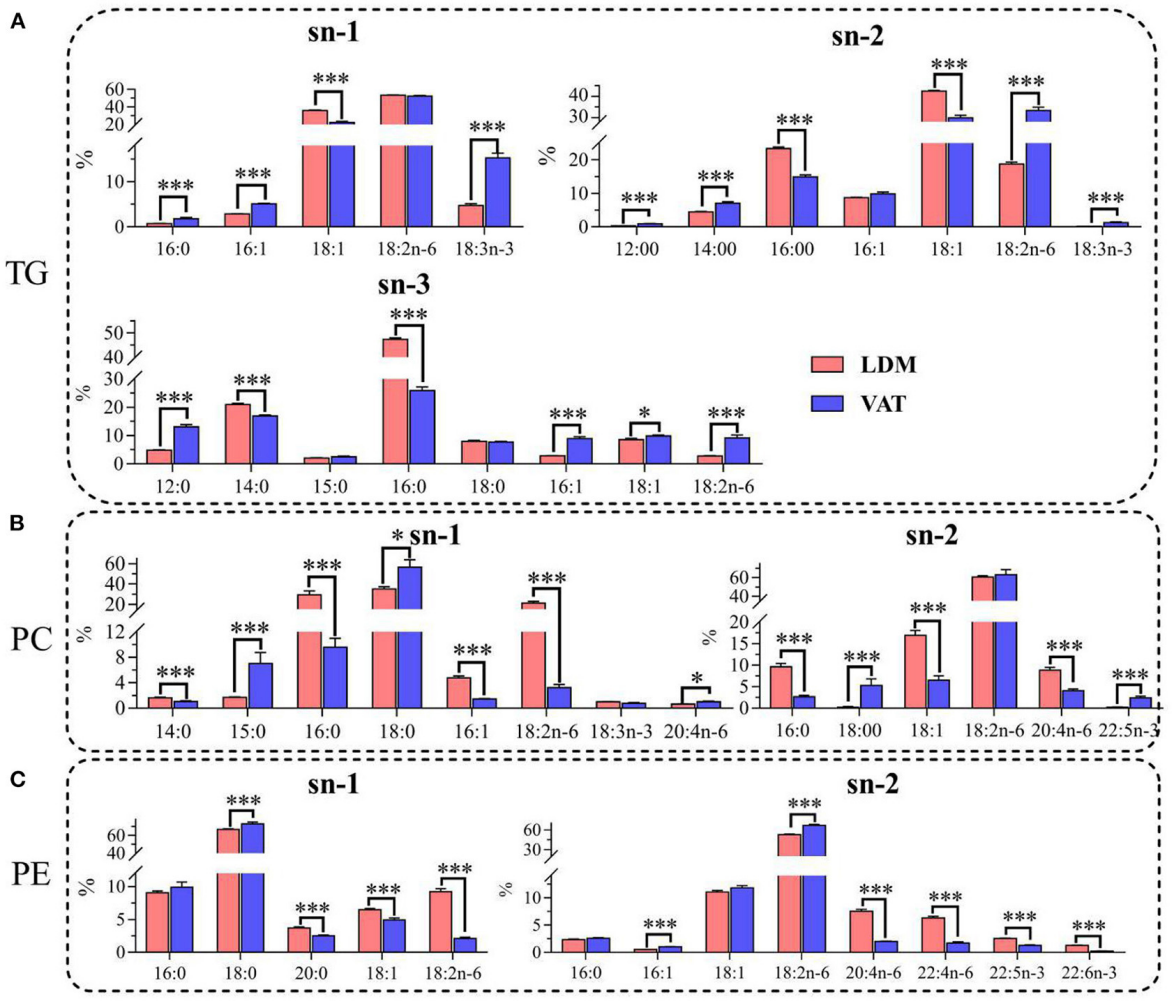
Positional distributions (sn-1, sn-2, and sn-3) of fatty acids in **(A)** TGs, **(B)** PC, and **(C)** PE in LDM and VAT of Dezhou donkey. Values are presented as means ± SEM (*n* = 9), *p < 0.05, ***p < 0.001. TG, triglyceride; PC, phosphatidylcholine; PE, phosphatidylethanolamine.

As shown in Figure 2, the percentage contents of saturated fatty acid (SFAs) (12:0, 14:0, 16:0, and 18:0) in TGs were higher at the sn-3 position than that at the sn-1 and sn-2 positions, while the percentage contents of SFAs in PC and PE were higher at the sn-1 position. Furthermore, the percentages of 16:0 at the sn-2 and sn-3 positions in TGs, and the sn-1 and sn-2 positions in PC, and 14:0 at the sn-3 positions in TGs were significantly higher in LDM than in VAT (*p* < 0.001). In contrast, the percentages of 18:0 at the two positions in PC and PE were significantly lower in LDM than in VAT (*p* < 0.001).

The percentage contents of monounsaturated fatty acid (MUFAs) (16:1 and 18:1) at the sn-2 positions of TGs, PC, and PE were higher than those at the sn-1 or sn-3 positions. The percentage contents of 18:1 at the sn-1 and/or sn-2 positions of TGs, PC, and PE were significantly higher in LDM than in VAT (*p* < 0.001), while the percentage contents of 16:1 and 18:1 at the sn-3 positions of TGs were higher in VAT than in LDM (*p* < 0.05).

The distributions of PUFAs (18:2n-6, 18:3n-3, and 20:4n-6) were higher at the sn-1 position in TGs, and at the sn-2 positions in PC and PE. Furthermore, the percentage contents of 18:2n-6 at the sn-2 and sn-3 positions in TGs, and the sn-2 position in PE, were significantly lower in LDM than in VAT, while percentage contents of 18:2n-6 at the sn-1 position in PC were higher in VAT than in LDM (*p* < 0.001). The percentage contents of 18:3n-3 at the sn-1 and sn-2 positions in TGs were significantly lower in LDM than in VAT (*p* < 0.001), while the percentage contents of 20:4n-6, 22:4n-6, 22:5n-3, and 22:6n-3 at the sn-1 and sn-2 positions in PC and PE were significantly higher in VAT than in LDM (*p* < 0.001).

### Differential Lipid Molecules and Potential Lipid Markers

**Figure 3:**
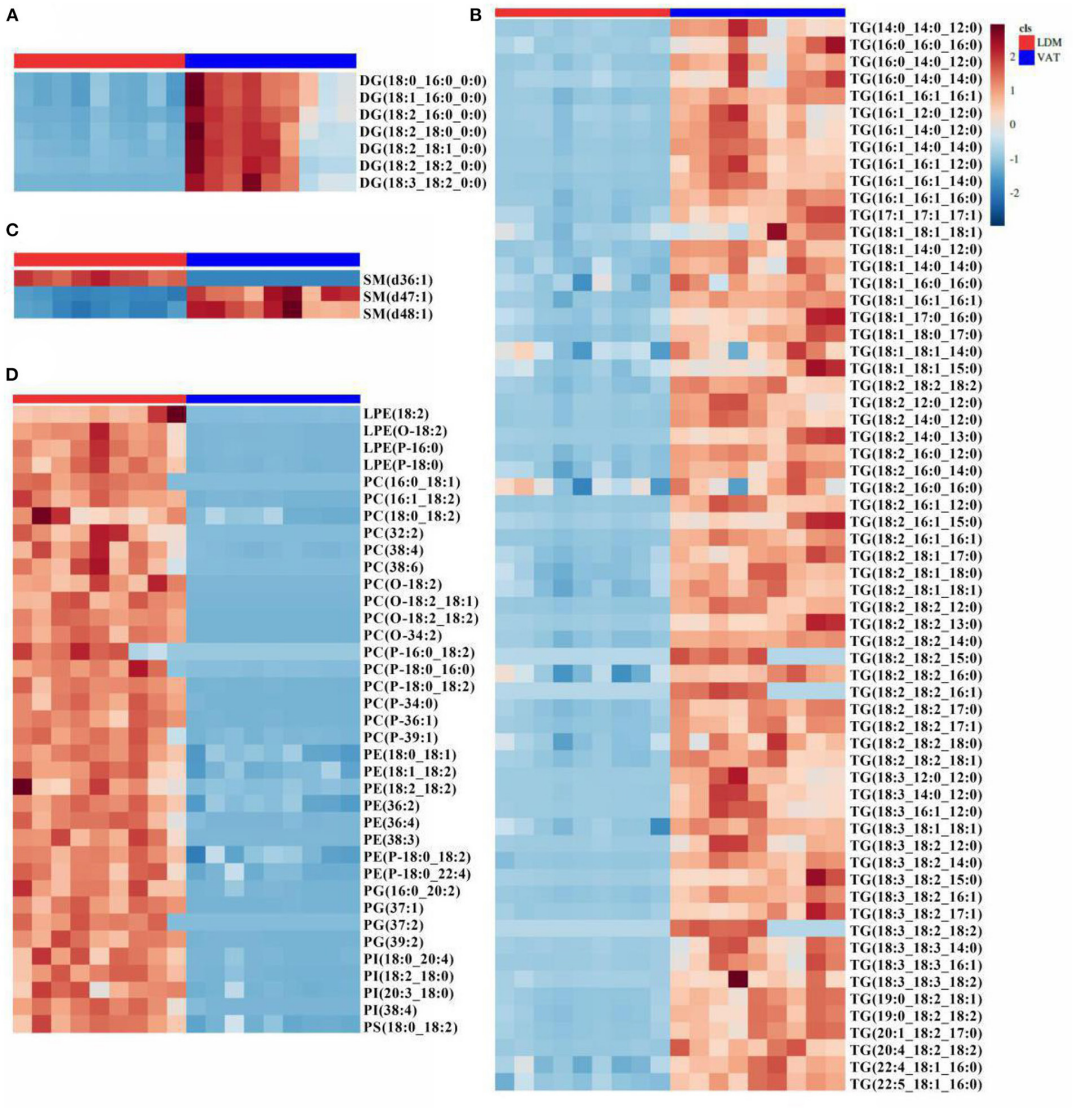
Difference in lipid molecules of donkey LDM and VAT. Heatmap analysis of **(A)** DG, **(B)** TG, **(C)** SP, and **(D)** GP molecules. Colors indicate decreased (blue band) or increased (red band) levels of lipid molecules in LDM vs. VAT. DG, diacylglycerol; TG, triglyceride; SP, sphingolipids; GP, glycerophospholipids.

As shown in Supplementary Figure S2, 110 lipids were identified as differential lipid molecules, of which 72 were downregulated and 38 were upregulated in LDM compared with VAT. These differential lipid molecules comprised 4 LPEs, 16 PCs, 8 PEs, 4 PGs, 4 PIs, 1 PS, 3 SMs, 7 DGs, and 63 TGs (Supplementary Table S1; |log2(Fold Change)| >1; VIP >1; *p* < 0.05). The 72 downregulated lipids comprised 7 DGs, 63 TGs, and 2 SMs, while the 38 upregulated lipids comprised 4 LPEs, 16 PCs, 8 PEs, 4 PGs, 4 PIs, 1 PS, and 1 SM (Figures 3A–D).

**Figure 4:**
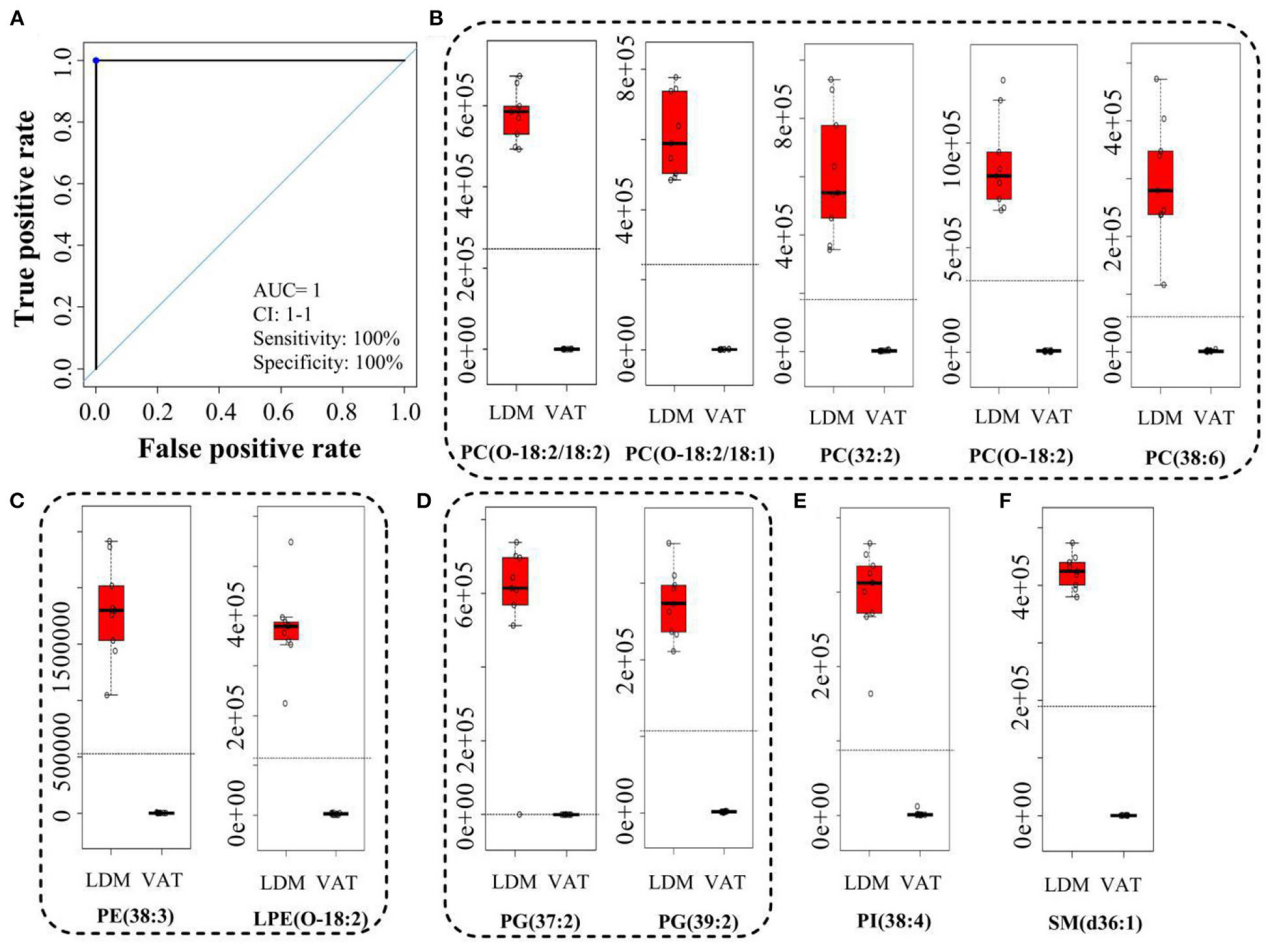
Receiver operating characteristic curve and normalized intensity for potential lipid markers. **(A)** Receiver operating characteristic curve of **(B–F)**. **(B–F)** Normalized intensity for potential lipid markers in LDM and VAT. AUC is the area under the ROC curve; CI 1-1 is the lower and upper limit of the AUC confidence interval.

Figure 4A and Supplementary Table S2 showed the receiver operating characteristic (ROC) curves and parameters for the top 11 discriminating lipids, with an area under the ROC curve (AUC) of 1, specificity of 100%, and sensitivity of 100% for PC(O-18:2/18:2), PC(O-18:2/18:1), PC(32:2), PC(O-18:2), PC(38:6), PE(38:3), LPE(O-18:2), PG(37:2), PG(39:2), PI(38:4), and SM(d36:1). These potential lipid markers comprised 5 PCs, 2 PEs, 2 PGs, 1 PI, and 1 SM, for which the normalized intensity was significantly higher in LDM than in VAT, and close to 0 in VAT (Figures 4B–F).

### Lipid Metabolism Pathways

**Figure 5:**
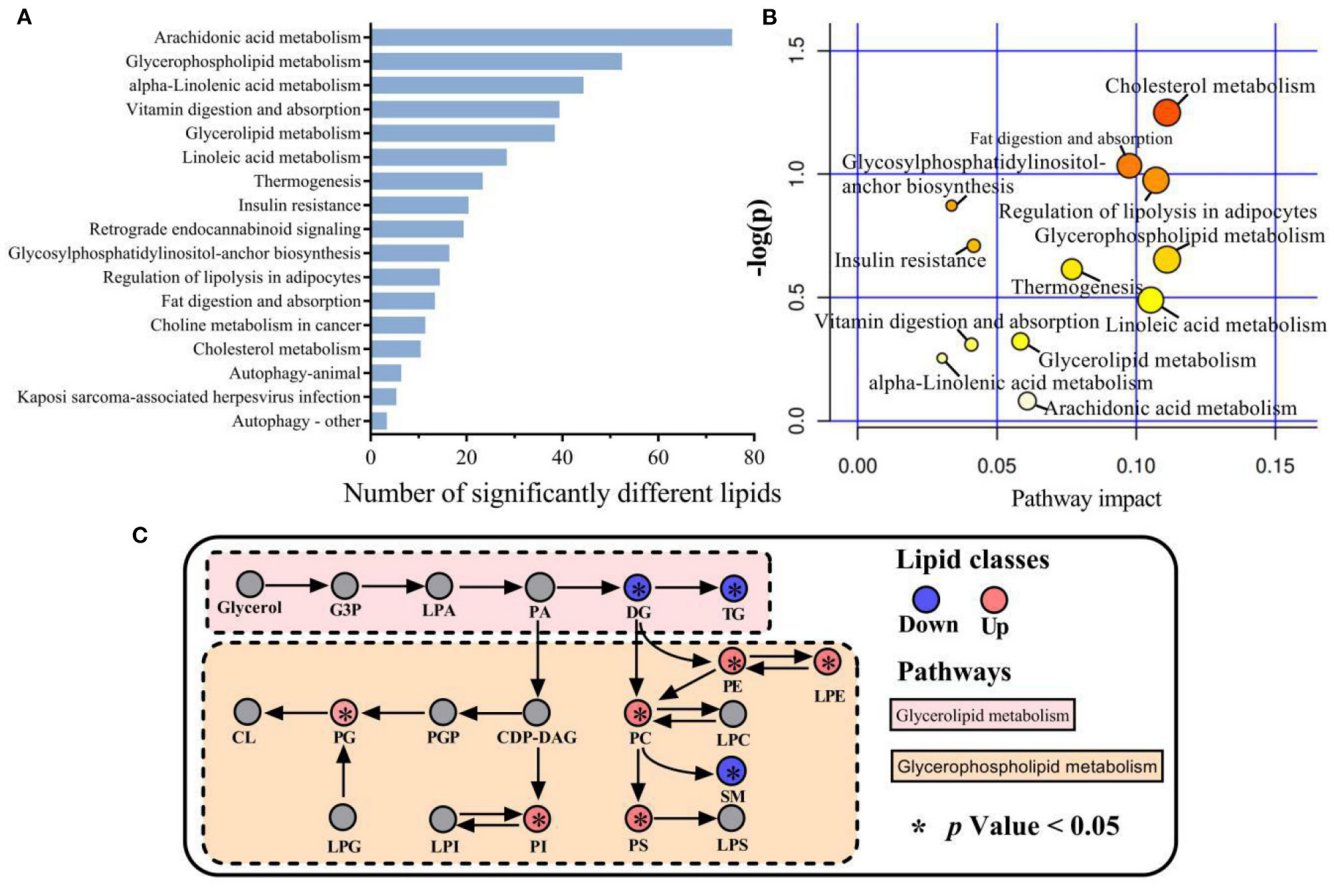
Metabolic pathways involved in different lipid species of LDM and VAT. **(A)** Kyoto Encyclopedia of Genes and Genomes (KEGG) enrichment pathways of significantly different lipids in LDM compared with VAT. **(B)** Map of significant metabolic pathways in LDM compared with VAT. **(C)** Enrichment of significantly different lipids in glycerolipid and glycerophospholipid metabolism in LDM compared with VAT.

Functional enrichment analysis was conducted on the 110 lipids showing significant differences between LDM and VAT using the KEGG pathways. This revealed significant enrichment of 17 major metabolic pathways, including arachidonic acid metabolism, GP metabolism, α-linolenic acid metabolism, vitamin digestion and absorption, GL metabolism, and linoleic acid metabolism (Figure 5A). Cholesterol metabolism, fat digestion and absorption, lipolysis regulation in adipocytes, GP metabolism, GL metabolism, thermogenesis, linoleic acid metabolism, arachidonic acid metabolism, linolenic acid metabolism, vitamin digestion and absorption, insulin resistance, and glycosylphosphatidylinositol-anchor biosynthesis were the most relevant metabolic pathways, as shown in Figure 5B. GL and GP metabolisms are shown in Figure 5C. The expression of DG and TGs by GL metabolism was significantly lower in LDM than in VAT (*p* < 0.05), while the expression of PC, PE, PS, PG, and LPE by GP metabolism was significantly lower in VAT than in LDM (*p* < 0.05).

### Gene Expression in Lipid Metabolic Pathways

As shown in Supplementary Table S3, 55,118,7981,721,661 and 56,402,6561,200,757 clean reads were obtained in LDM and VAT, respectively. After *de novo* assembly, 154,948,346 unigenes were obtained, with the GC percentage reaching 47.02%. The clean reads were matched with the corresponding assembly sequences, and the BUSCO score was 82.30%. The 138,936 assembled unigenes were annotated by the BLAST tool using six public databases. Among them, 20,587 (14.82%), 20,300 (15.61%), 24,000 (17.27%), 39,202 (28.22%), 26,119 (18.80%), and 17,537 (12.62%) unigenes were matched with the GO, KEGG, COG, NR, Swiss-Prot, and Pfam databases, respectively. A total of 40,696 (29.29%) unigenes were annotated in six public databases.

**Figure 6:**
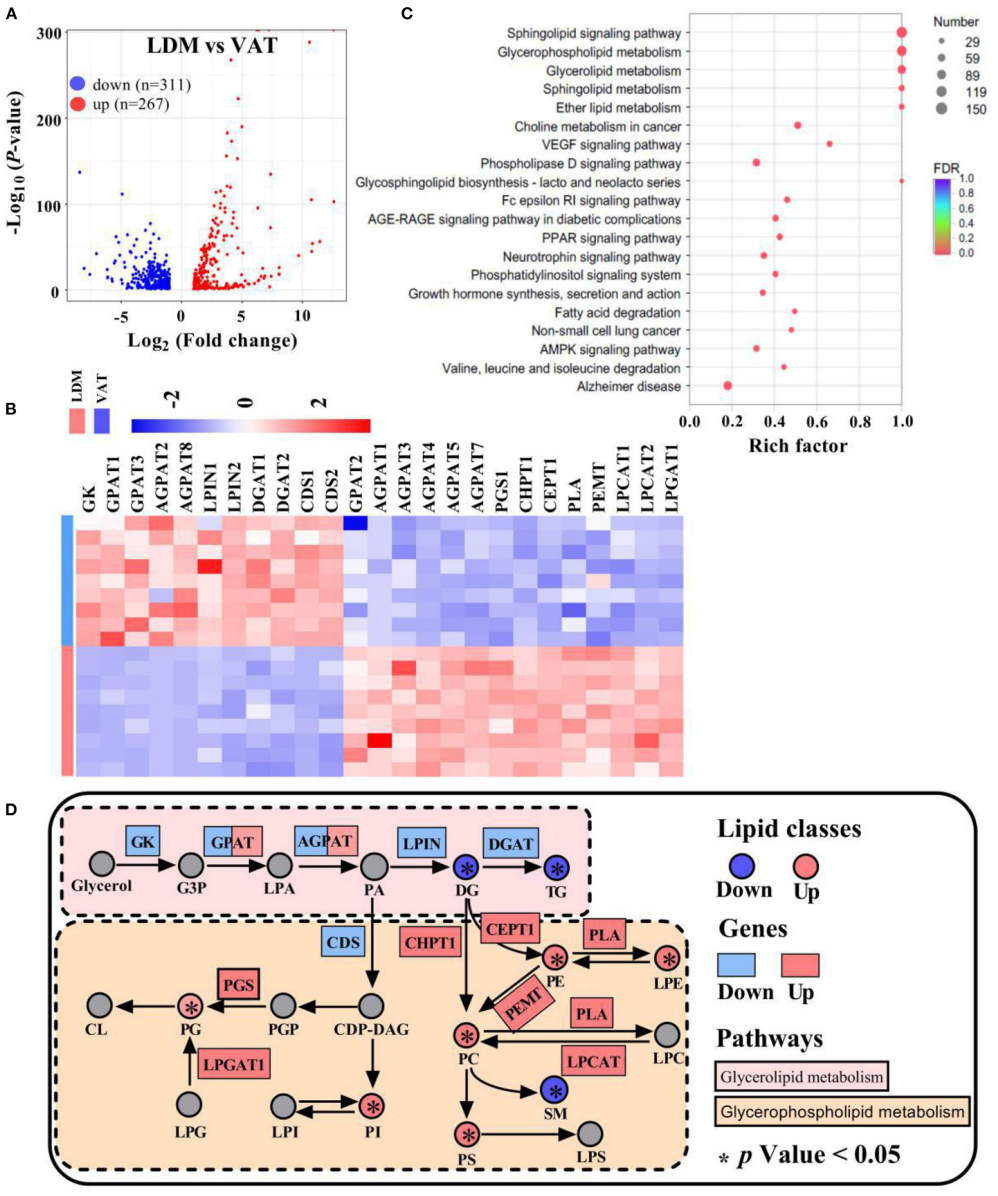
Expression of lipid metabolism-related genes in LDM and VAT. **(A)** Differential expressed genes between LDM and VAT. **(B)** Heatmap of relative expression of selected major lipid metabolism-related genes in LDM compared with VAT. **(C)** KEGG enrichment pathways of different lipid metabolism-related genes in LDM compared with VAT. **(D)** Selected glycerolipid and glycerophospholipid metabolisms reactions from KEGG, with indications of lipid classes and genes significantly regulated in LDM compared with VAT.

A total of 578 differentially expressed genes were identified, of which 311 were downregulated and 267 were upregulated in LDM compared with VAT (Figure 6A). RNA-seq analysis showed that the expression of GL and GP metabolism-related genes, including glycerol kinase (GK), GPAT1 and GPAT3, AGPAT2 and AGPAT8, LPIN1 and LPIN2, DGAT1 and DGAT2, and cytidylyltransferases 1 and 2 (CDS1 and CDS2), was significantly downregulated in LDM compared with VAT. Meanwhile, the expression of GPAT2, AGPAT1, AGPAT3, AGPAT4, AGPAT5, AGPAT7, phosphatidylglycerol 3-phosphatidyltransferase (PGS1), CHPT1, choline/ethanolaminephosphotransferase 1 (CEPT1), phospholipase (PLA), phosphatidylethanolamine N-methyltransferase (PEMT), LPCAT1 and LPCAT2, and lysophosphatidylglycerol acyltransferase (LPGAT1) was significantly upregulated in LDM compared with VAT (Figure 6B). KEGG enrichment analysis of the differentially expressed genes showed that genes regulating GL, GP, and sphingolipid metabolisms, and fatty acid degradation, were enriched (Figure 6C). Functional enrichment analysis of GL and GP metabolisms conducted using the KEGG pathways showed that the expression of GL metabolism-related genes was significantly decreased in LDM compared with VAT (*p* < 0.05), while the expression of GP metabolism-related genes was significantly increased in LDM compared with VAT (*p* < 0.05), as shown in Figure 6D.

## Discussion

In contrast to traditional approaches, LC-MS-based lipidomics allows simultaneous identification and quantification of more than 1,000 lipid molecules, which directly clarifies the interrelation between phenotype and mechanism (20). The heterogeneity of IMF and VAT was elucidated using lipidomics analysis, which provides better understanding of the muscle nutritional value and improve IMF content. In the present study, 1,146 and 1,134 lipids were identified in LDM and VAT, respectively, comprising 18 subclasses. These results were consistent with the 1,180 and 1,127 lipid species found in pork (15) and chicken (16), respectively, and significantly higher than found in donkey milk (335 lipids species) (13) in previous studies. Furthermore, the number and relative content of GPs (such as PC, PE, PG, PI, and PS) and SL (SM) were significantly higher in LDM than in VAT. These findings further confirmed that IMF was rich in lipid classes, especially phospholipid classes (4). Furthermore, TGs were identified as the predominant lipid class in LDM, followed by PC and PE, which were selected for more intensive analysis.

In the present study, the SFAs (14:0, 16:0, and 18:0) in donkey muscle were distributed preferentially at the outer positions of the glycerol backbone in predominant lipid classes (sn-3 positions of TG molecules and sn-1 positions of PC and PE molecules). These results were in agreement with those reported for Nile tilapia filet (21). However, 16:0 is distributed preferentially at the sn-2 position in breast, bovine milk, and lard, which are easily digested and absorbed by humans, with an excess intake of 16:0 at the sn-2 position of lipids potentially increasing the risk of obesity and atherogenesis (22, 23). In contrast, 16:0 at the sn-1/3 positions of lipids are selectively lipolyzed by pancreatic lipase (24). This result suggested that, after human consumption, SFAs in donkey meat are easily mobilized and consumed, reducing the chance of fat storage and risk of cardiovascular and cerebrovascular diseases, hypertension, and obesity in humans. In this study, 18:1 fatty acids were esterified preferentially at the 2-position of TGs, PC, and PE. This result was consistent with previously reported 18:1 fatty acids in vegetable and animal (most non-milk) lipids (25). The percentage contents of 18:1 in TGs, PC, and PE in LDM were significantly higher than those in VAT. As 18:1 has the ability to remove bad cholesterol and protect cardiovascular and cerebrovascular health in human nutrition (26), the nutritional value of donkey meat needs further investigation. This result suggested that donkey meat is a good source of 18:1 for human consumption.

The present study showed that PUFAs were preferentially deposited at the sn-1 positions in TGs, and the sn-2 positions in PC and PE. Regarding nutrition, 18:2n-6 at the sn-1/3 positions is more likely to be released from TGs into the circulation system, and might increase the risk of inflammation (27). A recent study showed that, in fish fed with perilla oil and fish oil enriched with n-3 PUFAs, the levels of 18:2n-6 at the sn-2 position in TGs in the filets was increased compared with those fed with palm oil, olive oil, and safflower oil (21), indicating that the positional distribution of 18:2n-6 in TGs was affected by dietary oils. In PC and PE molecules, PUFAs were preferentially deposited at the sn-2 position, which is generally consistent with previous studies, and is relatively stable and better retained to play roles in important functions (25, 28). Furthermore, the PUFA levels in PC and PE were significantly higher in LDM than in VAT. These results further demonstrated that IMF was rich in PUFAs, especially at the sn-2 position.

In the present study, the difference between IMF and VAT at the lipid molecular level was mainly manifested in GPs (PC, PE, LPE, PG, PI, PS) and GLs (DG and TG), with IMF rich in GPs and VAT rich in GLs. This result was consistent with a previous report, which showed that the LDM of pigs contains a large GP content and upregulated PUFAs (29). The heterogeneity of lipid profiles in IMF and VAT could be associated with cell type and fat deposition rates (30). GPs, which show significant differences among different types of cell, are the main components of cell membrane, where they play key roles, such as providing structural attributes and signaling processes (31, 32). IMF was rich in GPs due to originating from muscle cells and adipocytes, while VAT only originated from adipocytes. Furthermore, IMF exhibits smaller adipocyte diameters and lower adipose maturation compared with VAT (33). A higher proportion of energy is available for fat in muscle, and the content of IMF (only 2–5% on average) might be lower than in other adipose tissues (34). In this study, the above lipids were enriched in 17 metabolic pathways. According to the degree of impact on the metabolic pathway, GL and GP metabolisms were key metabolic pathways regulating lipid deposition. This result was similar to the findings reported for IMF deposition in pork (29). This was further supported by the GLs and GPs of significantly different lipids being mostly synthesized by the TG and GP pathways (9).

Lipid accumulation can be regulated by transcription, leading to different lipid profiles in tissues (35). Previous studies have shown that the differential deposition of IMF in pork might be caused by differences in fatty acid, GL, and GP metabolism pathways (29, 36). In the present study, the transcriptome results showed that, among a total of 578 differentially expressed genes were screened, the expression of GL metabolism-related genes was significantly downregulated in LDM compared with VAT, while the opposite trend was observed for the expression of GP metabolism-related genes. Furthermore, functional enrichment analysis revealed the differentially expressed genes involved in the GL and GP metabolism, and PPARs and AMPK signaling pathways. These findings indicated that the GL and GP metabolism pathways were key pathways in regulating IMF at the molecular level. Indeed, this was supported by the lipid being mainly composed of TGs, and phospholipids being synthesized by the GL and GP pathways (9).

## Conclusion

This study analyzed lipid profiles and metabolism in IMF and VAT from donkeys using non-targeted lipidomics and transcriptomics. A total of 1,146 and 1,134 lipids were identified in LDM and VAT, respectively. Donkey IMF is rich in GPs and PUFAs distributed preferentially at the sn-1 positions of TGs and sn-2 positions of PC and PE. This phenotype might result from the higher content of 38 lipid molecules (37 GPs and 1 SM) and the expression of lipid synthesis-related genes in IMF. GL and GP metabolisms were considered key IMF-regulating pathways. These results provide new perspectives for understanding the heterogeneity of IMF and VAT, and developing new strategies to regulate IMF deposition.

## Data Availability Statement

The datasets presented in this study can be found in online repositories. The names of the repository/repositories and accession number(s) can be found below: BioProject's metadata is available at https://dataview.ncbi.nlm.nih.gov/object/PRJNA751405?reviewer=rcmbutjn0mfgjtrqeam5sqejne.

## Ethics Statement

The animal study was reviewed and approved by Liaocheng University Animal Care and Use Committee.

## Author Contributions

ML: conceptualization, methodology, formal analysis, investigation, and writing (original draft and editing). MZ and WC: investigation, formal analysis, writing (review and editing), and validation. YW: software and formal analysis. YinghS, BL, and CC: investigation and formal analysis. YingzS, XS, and PX: investigation and resources. CW: supervision, writing (review and editing), and funding acquisition. All authors contributed to the article and approved the submitted version.

## Funding

This work was supported by the Open Project of Liaocheng University Animal Husbandry Discipline (319312101-10), the Open Project of Shandong Collaborative Innovation Center for Donkey Industry Technology (3193308), the Scientific Research Fund of Liaocheng University (318052019), the Innovation and Entrepreneurship Training Program for College Students (202110447017 and CXCY2021002), the Well-bred Program of Shandong Province (2017LZGC020), the Shandong Province Modern Agricultural Technology System Donkey Industrial Innovation Team (SDAIT-27), and the Taishan Leading Industry Talents-Agricultural Science of Shandong Province (LJNY201713).

## Conflict of Interest

The authors declare that the research was conducted in the absence of any commercial or financial relationships that could be construed as a potential conflict of interest.

## Publisher's Note

All claims expressed in this article are solely those of the authors and do not necessarily represent those of their affiliated organizations, or those of the publisher, the editors and the reviewers. Any product that may be evaluated in this article, or claim that may be made by its manufacturer, is not guaranteed or endorsed by the publisher.
