# Supplementary material for: Determination of the Heterogeneity of Intramuscular Fat and Visceral Adipose Tissue From Dezhou Donkey by Lipidomics and Transcriptomics Profiling
Source: Front Nutr. 2021 Sep 28;8:746684. doi: 10.3389/fnut.2021.746684 (PMC8505748; doi:10.3389/fnut.2021.746684)
Supplement: Supplementary file 1 [file Data_Sheet_1.doc]

**Supplemental file**

**
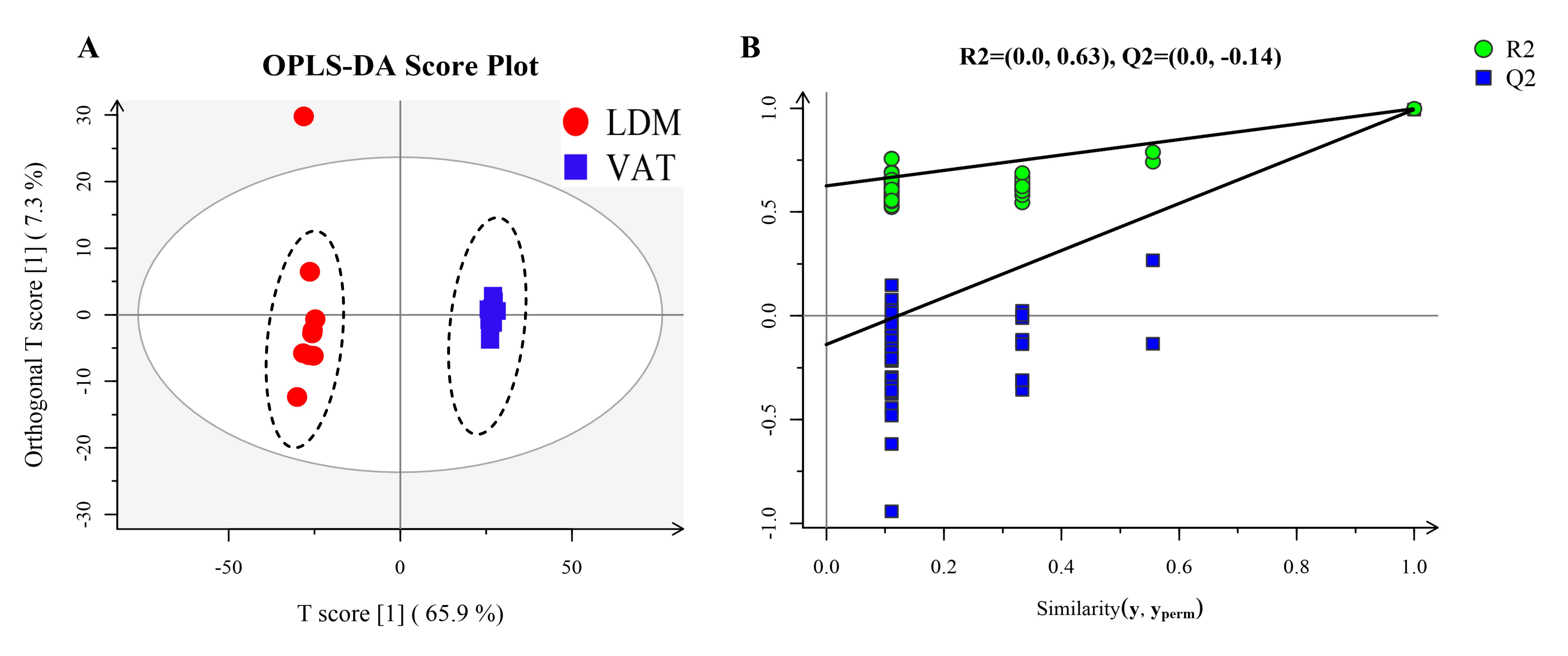
**

**Fig. S1** (A) OPLS-DA score plots are based on lipidomic data from intramuscular fat and visceral adipose tissue (*R*^2^*X* = 0.863, *R*^2^*Y* = 0.993, *Q*^2^ = 0.892) and (B) corresponding OPLS-DA validation plots (*R*^2^ = (0.0, 0.63), *Q*^2^ = (0.0, -0.11).

**
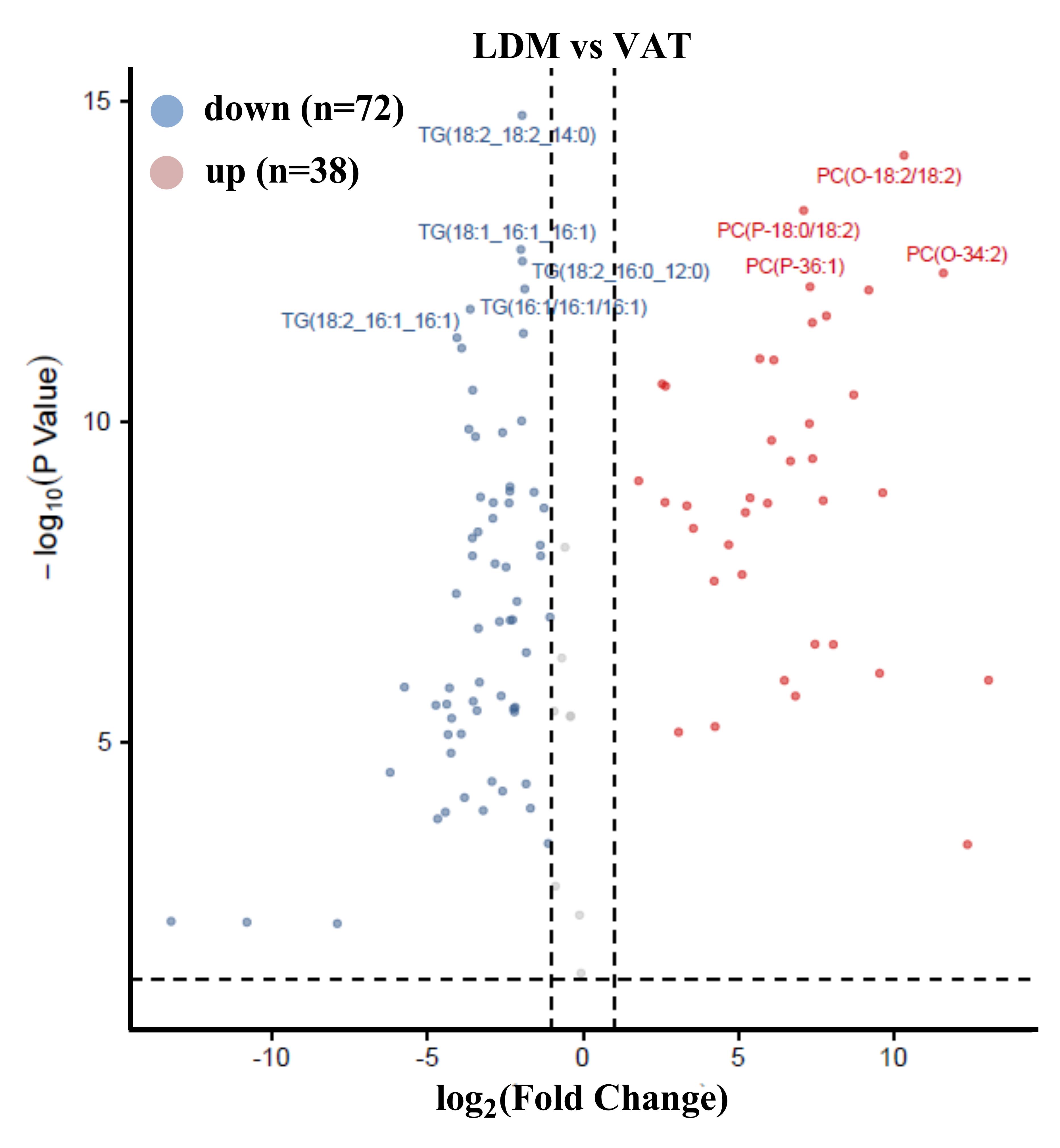
**

**Fig. S2** Log_2_ (fold changes) in lipid molecule in LDM and VAT and the corresponding significance values displayed as -log10 (*P* value). The transverse and vertical dotted lines indicate the cutoff value for differential expression (*P* < 0.05 and |Log_2_ (fold changes)| > 1). In total, 38 and 72 lipid molecules were identified that had up-regulation (red) or down-regulation (blue) levels in LDM vs. VAT.

**Table S1 Information on different lipid molecules in LDM and VAT of Dezhou donkey**

| No. | Lipid name | Lipid category | Accurate mass | Rt (s) | Fold Change | log2(FC) | *P* value | FDR | VIP |
| --- | --- | --- | --- | --- | --- | --- | --- | --- | --- |
| 1 | LPE(18:2) | GPs | 476.2768 | 71.2530 | 88.64 | 6.47 | 0.0000 | 0.0000 | 1.26 |
| 2 | LPE(O-18:2) | GPs | 462.2978 | 100.5735 | 165.91 | 7.37 | 0.0000 | 0.0000 | 1.50 |
| 3 | LPE(P-16:0) | GPs | 436.2826 | 95.8190 | 101.29 | 6.66 | 0.0000 | 0.0000 | 1.10 |
| 4 | LPE(P-18:0) | GPs | 464.3132 | 132.1655 | 66.51 | 6.06 | 0.0000 | 0.0000 | 1.33 |
| 5 | PC(16:0_18:1) | GPs | 804.5573 | 401.1870 | 740.04 | 9.53 | 0.0000 | 0.0000 | 1.62 |
| 6 | PC(16:1_18:2) | GPs | 800.5363 | 362.6660 | 69.91 | 6.13 | 0.0000 | 0.0000 | 1.13 |
| 7 | PC(18:0_18:2) | GPs | 830.5805 | 463.2205 | 8.39 | 3.07 | 0.0000 | 0.0000 | 2.36 |
| 8 | PC(32:2) | GPs | 730.5302 | 352.3390 | 263.53 | 8.04 | 0.0000 | 0.0000 | 1.87 |
| 9 | PC(38:4) | GPs | 810.5990 | 423.4610 | 34.50 | 5.11 | 0.0000 | 0.0000 | 1.28 |
| 10 | PC(38:6) | GPs | 806.5693 | 359.7590 | 175.08 | 7.45 | 0.0000 | 0.0000 | 1.30 |
| 11 | PC(O-18:2) | GPs | 564.3288 | 73.7950 | 210.17 | 7.72 | 0.0000 | 0.0000 | 2.32 |
| 12 | PC(O-18:2_18:1) | GPs | 814.5854 | 446.1380 | 415.07 | 8.70 | 0.0000 | 0.0000 | 1.94 |
| 13 | PC(O-18:2_18:2) | GPs | 812.5716 | 437.5335 | 1273.90 | 10.32 | 0.0000 | 0.0000 | 1.90 |
| 14 | PC(O-34:2) | GPs | 788.5659 | 439.9990 | 3059.40 | 11.58 | 0.0000 | 0.0000 | 2.39 |
| 15 | PC(P-16:0_18:2) | GPs | 786.5560 | 430.5520 | 5237.40 | 12.36 | 0.0004 | 0.0006 | 2.15 |
| 16 | PC(P-18:0_16:0) | GPs | 790.5844 | 491.8020 | 113.30 | 6.82 | 0.0000 | 0.0000 | 1.15 |
| 17 | PC(P-18:0_18:2) | GPs | 814.5853 | 493.3990 | 136.05 | 7.09 | 0.0000 | 0.0000 | 1.91 |
| 18 | PC(P-34:0) | GPs | 746.5940 | 446.4045 | 225.59 | 7.82 | 0.0000 | 0.0000 | 1.85 |
| 19 | PC(P-36:1) | GPs | 816.6008 | 505.7020 | 156.85 | 7.29 | 0.0000 | 0.0000 | 1.42 |
| 20 | PC(P-39:1) | GPs | 858.6673 | 540.9930 | 25.58 | 4.68 | 0.0000 | 0.0000 | 1.25 |
| 21 | PE(18:0_18:1) | GPs | 744.5380 | 459.8800 | 5.82 | 2.54 | 0.0000 | 0.0000 | 1.09 |
| 22 | PE(18:1_18:2) | GPs | 740.5141 | 413.3360 | 6.19 | 2.63 | 0.0000 | 0.0000 | 1.07 |
| 23 | PE(18:2_18:2) | GPs | 738.5014 | 373.1055 | 18.89 | 4.24 | 0.0000 | 0.0000 | 1.01 |
| 24 | PE(36:2) | GPs | 742.5301 | 459.9010 | 6.27 | 2.65 | 0.0000 | 0.0000 | 3.50 |
| 25 | PE(36:4) | GPs | 740.5224 | 371.0800 | 60.95 | 5.93 | 0.0000 | 0.0000 | 1.06 |
| 26 | PE(38:3) | GPs | 770.5518 | 447.1530 | 791.33 | 9.63 | 0.0000 | 0.0000 | 3.29 |
| 27 | PE(P-18:0_18:2) | GPs | 726.5350 | 482.2770 | 3.45 | 1.78 | 0.0000 | 0.0000 | 3.03 |
| 28 | PE(P-18:0_22:4) | GPs | 778.5678 | 501.3170 | 11.64 | 3.54 | 0.0000 | 0.0000 | 1.11 |
| 29 | PG(16:0_20:2) | GPs | 773.5268 | 375.7060 | 41.22 | 5.37 | 0.0000 | 0.0000 | 1.94 |
| 30 | PG(37:1) | GPs | 789.5724 | 442.1090 | 580.13 | 9.18 | 0.0000 | 0.0000 | 1.56 |
| 31 | PG(37:2) | GPs | 787.5604 | 430.5310 | 8373.60 | 13.03 | 0.0000 | 0.0000 | 1.78 |
| 32 | PG(39:2) | GPs | 815.5897 | 446.3585 | 165.00 | 7.37 | 0.0000 | 0.0000 | 1.30 |
| 33 | PI(18:0_20:4) | GPs | 885.5395 | 351.6230 | 37.19 | 5.22 | 0.0000 | 0.0000 | 2.49 |
| 34 | PI(18:2_18:0) | GPs | 861.5409 | 363.4630 | 51.17 | 5.68 | 0.0000 | 0.0000 | 1.89 |
| 35 | PI(20:3_18:0) | GPs | 887.5512 | 376.6240 | 18.54 | 4.21 | 0.0000 | 0.0000 | 1.87 |
| 36 | PI(38:4) | GPs | 904.5915 | 357.9780 | 154.31 | 7.27 | 0.0000 | 0.0000 | 1.35 |
| 37 | PS(18:0_18:2) | GPs | 786.5197 | 376.5580 | 10.08 | 3.33 | 0.0000 | 0.0000 | 3.10 |
| 38 | SM(d36:1) | SPs | 775.5859 | 431.4550 | 1477.60 | 10.53 | 0.0000 | 0.0000 | 1.63 |
| 39 | SM(d47:1) | SPs | 885.7682 | 686.3400 | 0.08 | -3.67 | 0.0000 | 0.0000 | 2.09 |
| 40 | SM(d48:1) | SPs | 899.7778 | 679.5890 | 0.20 | -2.35 | 0.0000 | 0.0000 | 1.62 |
| 41 | DG(18:0_16:0_0:0) | GLs | 614.5658 | 529.8015 | 0.21 | -2.24 | 0.0000 | 0.0000 | 1.04 |
| 42 | DG(18:1_16:0_0:0) | GLs | 612.5572 | 529.8015 | 0.22 | -2.21 | 0.0000 | 0.0000 | 4.05 |
| 43 | DG(18:2_16:0_0:0) | GLs | 610.5414 | 497.8680 | 0.09 | -3.42 | 0.0000 | 0.0000 | 6.14 |
| 44 | DG(18:2_18:0_0:0) | GLs | 638.5727 | 533.3275 | 0.13 | -2.93 | 0.0000 | 0.0001 | 3.27 |
| 45 | DG(18:2_18:1_0:0) | GLs | 636.5560 | 502.1700 | 0.07 | -3.82 | 0.0001 | 0.0001 | 3.84 |
| 46 | DG(18:2_18:2_0:0) | GLs | 634.5394 | 468.6820 | 0.04 | -4.68 | 0.0002 | 0.0002 | 3.16 |
| 47 | DG(18:3_18:2_0:0) | GLs | 632.5234 | 434.4805 | 0.01 | -6.21 | 0.0000 | 0.0000 | 1.30 |
| 48 | TG(14:0_14:0_12:0) | GLs | 712.6443 | 640.1155 | 0.16 | -2.64 | 0.0000 | 0.0000 | 1.56 |
| 49 | TG(16:0_16:0_16:0) | GLs | 824.7598 | 730.5325 | 0.11 | -3.21 | 0.0001 | 0.0002 | 3.38 |
| 50 | TG(16:0_14:0_12:0) | GLs | 740.6746 | 665.2735 | 0.20 | -2.35 | 0.0000 | 0.0000 | 2.12 |
| 51 | TG(16:0_14:0_14:0) | GLs | 768.7064 | 687.6620 | 0.31 | -1.69 | 0.0001 | 0.0002 | 2.09 |
| 52 | TG(16:1_16:1_16:1) | GLs | 818.7135 | 670.5850 | 0.27 | -1.88 | 0.0000 | 0.0000 | 6.27 |
| 53 | TG(16:1_12:0_12:0) | GLs | 710.6285 | 615.6605 | 0.22 | -2.19 | 0.0000 | 0.0000 | 1.61 |
| 54 | TG(16:1_14:0_12:0) | GLs | 738.6598 | 641.9630 | 0.21 | -2.27 | 0.0000 | 0.0000 | 2.59 |
| 55 | TG(16:1_14:0_14:0) | GLs | 766.6819 | 645.0450 | 0.13 | -2.90 | 0.0000 | 0.0000 | 1.67 |
| 56 | TG(16:1_16:1_12:0) | GLs | 764.6656 | 622.1550 | 0.10 | -3.37 | 0.0000 | 0.0000 | 1.02 |
| 57 | TG(16:1_16:1_14:0) | GLs | 792.6994 | 647.5890 | 0.10 | -3.30 | 0.0000 | 0.0000 | 1.76 |
| 58 | TG(16:1_16:1_16:0) | GLs | 820.7301 | 670.6070 | 0.26 | -1.92 | 0.0000 | 0.0000 | 2.22 |
| 59 | TG(17:1_17:1_17:1) | GLs | 860.7603 | 703.8460 | 0.28 | -1.83 | 0.0000 | 0.0000 | 3.51 |
| 60 | TG(18:1_18:1_18:1) | GLs | 902.8157 | 737.3965 | 0.54 | -0.89 | 0.0017 | 0.0023 | 4.04 |
| 61 | TG(18:1_14:0_12:0) | GLs | 766.6911 | 666.1965 | 0.25 | -1.98 | 0.0000 | 0.0000 | 4.21 |
| 62 | TG(18:1_14:0_14:0) | GLs | 794.7147 | 687.9840 | 0.62 | -0.69 | 0.0000 | 0.0000 | 2.85 |
| 63 | TG(18:1_16:0_16:0) | GLs | 850.7766 | 731.8000 | 0.75 | -0.41 | 0.0000 | 0.0000 | 3.15 |
| 64 | TG(18:1_16:1_16:1) | GLs | 846.7358 | 673.6590 | 0.25 | -2.00 | 0.0000 | 0.0000 | 2.62 |
| 65 | TG(18:1_17:0_16:0) | GLs | 864.7991 | 745.8970 | 0.28 | -1.84 | 0.0000 | 0.0001 | 2.53 |
| 66 | TG(18:1_18:0_17:0) | GLs | 892.8208 | 746.8950 | 0.18 | -2.48 | 0.0000 | 0.0000 | 1.68 |
| 67 | TG(18:1_18:1_14:0) | GLs | 848.7699 | 711.5900 | 0.92 | -0.12 | 0.0049 | 0.0061 | 1.71 |
| 68 | TG(18:1_18:1_15:0) | GLs | 862.7834 | 723.5100 | 0.46 | -1.12 | 0.0004 | 0.0005 | 2.69 |
| 69 | TG(18:2_18:2_18:2) | GLs | 896.7585 | 680.1540 | 0.20 | -2.36 | 0.0000 | 0.0000 | 8.69 |
| 70 | TG(18:2_12:0_12:0) | GLs | 736.6439 | 619.3650 | 0.16 | -2.69 | 0.0000 | 0.0000 | 2.57 |
| 71 | TG(18:2_14:0_12:0) | GLs | 764.6757 | 645.0535 | 0.14 | -2.89 | 0.0000 | 0.0000 | 4.82 |
| 72 | TG(18:2_14:0_13:0) | GLs | 778.6892 | 656.4145 | 0.09 | -3.54 | 0.0000 | 0.0000 | 1.18 |
| 73 | TG(18:2_16:0_12:0) | GLs | 792.7070 | 668.0085 | 0.26 | -1.95 | 0.0000 | 0.0000 | 5.72 |
| 74 | TG(18:2_16:0_14:0) | GLs | 820.7378 | 690.3970 | 0.67 | -0.58 | 0.0000 | 0.0000 | 3.94 |
| 75 | TG(18:2_16:0_16:0) | GLs | 848.7602 | 710.7360 | 0.95 | -0.07 | 0.0394 | 0.0451 | 1.16 |
| 76 | TG(18:2_16:1_12:0) | GLs | 790.6908 | 647.9090 | 0.10 | -3.38 | 0.0000 | 0.0000 | 5.03 |
| 77 | TG(18:2_16:1_15:0) | GLs | 832.7361 | 683.1260 | 0.17 | -2.59 | 0.0001 | 0.0001 | 1.86 |
| 78 | TG(18:2_16:1_16:1) | GLs | 844.7277 | 654.6980 | 0.08 | -3.63 | 0.0000 | 0.0000 | 2.22 |
| 79 | TG(18:2_18:1_17:0) | GLs | 888.7999 | 725.2380 | 0.23 | -2.13 | 0.0000 | 0.0000 | 4.12 |
| 80 | TG(18:2_18:1_18:0) | GLs | 902.8042 | 745.1630 | 0.39 | -1.37 | 0.0000 | 0.0000 | 6.40 |
| 81 | TG(18:2_18:1_18:1) | GLs | 900.7813 | 700.7115 | 0.42 | -1.26 | 0.0000 | 0.0000 | 2.68 |
| 82 | TG(18:2_18:2_12:0) | GLs | 816.7061 | 651.2440 | 0.09 | -3.55 | 0.0000 | 0.0000 | 5.38 |
| 83 | TG(18:2_18:2_13:0) | GLs | 830.7191 | 663.5045 | 0.07 | -3.92 | 0.0000 | 0.0000 | 1.31 |
| 84 | TG(18:2_18:2_14:0) | GLs | 844.7367 | 674.1880 | 0.26 | -1.96 | 0.0000 | 0.0000 | 6.98 |
| 85 | TG(18:2_18:2_15:0) | GLs | 858.7434 | 684.9465 | 0.00 | -7.90 | 0.0066 | 0.0081 | 2.00 |
| 86 | TG(18:2_18:2_16:0) | GLs | 872.7587 | 695.7310 | 0.75 | -0.41 | 0.0000 | 0.0000 | 3.85 |
| 87 | TG(18:2_18:2_16:1) | GLs | 870.7430 | 677.3020 | 0.00 | -10.81 | 0.0063 | 0.0077 | 4.73 |
| 88 | TG(18:2_18:2_17:0) | GLs | 886.7837 | 705.3750 | 0.17 | -2.59 | 0.0000 | 0.0000 | 3.81 |
| 89 | TG(18:2_18:2_17:1) | GLs | 884.7588 | 686.0960 | 0.09 | -3.46 | 0.0000 | 0.0000 | 2.82 |
| 90 | TG(18:2_18:2_18:0) | GLs | 900.8006 | 717.3635 | 0.52 | -0.93 | 0.0000 | 0.0000 | 4.87 |
| 91 | TG(18:2_18:2_18:1) | GLs | 898.7749 | 679.5970 | 0.19 | -2.38 | 0.0000 | 0.0000 | 3.57 |
| 92 | TG(18:3_12:0_12:0) | GLs | 734.6284 | 594.7420 | 0.04 | -4.74 | 0.0000 | 0.0000 | 1.58 |
| 93 | TG(18:3_14:0_12:0) | GLs | 762.6591 | 622.5000 | 0.10 | -3.33 | 0.0000 | 0.0000 | 2.79 |
| 94 | TG(18:3_16:1_12:0) | GLs | 788.6739 | 626.4770 | 0.05 | -4.38 | 0.0000 | 0.0000 | 2.74 |
| 95 | TG(18:3_18:1_18:1) | GLs | 898.7840 | 699.3520 | 0.34 | -1.58 | 0.0000 | 0.0000 | 6.85 |
| 96 | TG(18:3_18:2_12:0) | GLs | 814.6905 | 631.4940 | 0.05 | -4.23 | 0.0000 | 0.0000 | 2.02 |
| 97 | TG(18:3_18:2_14:0) | GLs | 842.7212 | 655.3470 | 0.06 | -4.06 | 0.0000 | 0.0000 | 6.05 |
| 98 | TG(18:3_18:2_15:0) | GLs | 856.7352 | 667.0590 | 0.05 | -4.25 | 0.0000 | 0.0000 | 1.59 |
| 99 | TG(18:3_18:2_16:1) | GLs | 868.7381 | 658.1890 | 0.07 | -3.91 | 0.0000 | 0.0000 | 6.12 |
| 100 | TG(18:3_18:2_17:1) | GLs | 882.7481 | 669.7355 | 0.05 | -4.30 | 0.0000 | 0.0000 | 1.26 |
| 101 | TG(18:3_18:2_18:2) | GLs | 894.7431 | 661.8040 | 0.00 | -13.25 | 0.0061 | 0.0075 | 4.85 |
| 102 | TG(18:3_18:3_14:0) | GLs | 840.7064 | 635.8275 | 0.05 | -4.34 | 0.0000 | 0.0000 | 1.79 |
| 103 | TG(18:3_18:3_16:1) | GLs | 866.7222 | 639.8910 | 0.02 | -5.75 | 0.0000 | 0.0000 | 1.67 |
| 104 | TG(18:3_18:3_18:2) | GLs | 892.7543 | 642.9060 | 0.05 | -4.43 | 0.0001 | 0.0002 | 3.18 |
| 105 | TG(19:0_18:2_18:1) | GLs | 916.8192 | 750.2440 | 0.09 | -3.56 | 0.0000 | 0.0000 | 2.40 |
| 106 | TG(19:0_18:2_18:2) | GLs | 914.8140 | 727.0000 | 0.14 | -2.83 | 0.0000 | 0.0000 | 1.48 |
| 107 | TG(20:1_18:2_17:0) | GLs | 916.8300 | 747.7640 | 0.09 | -3.56 | 0.0000 | 0.0000 | 2.38 |
| 108 | TG(20:4_18:2_18:2) | GLs | 920.7546 | 653.8450 | 0.06 | -4.08 | 0.0000 | 0.0000 | 1.30 |
| 109 | TG(22:4_18:1_16:0) | GLs | 926.8029 | 725.2610 | 0.48 | -1.07 | 0.0000 | 0.0000 | 1.45 |
| 110 | TG(22:5_18:1_16:0) | GLs | 924.7989 | 706.8010 | 0.38 | -1.38 | 0.0000 | 0.0000 | 1.74 |

Rt, retention time; FDR, false discovery rate; VIP, variable importance in projection; GPs, glycerophospholipids; GLs, glycerolipids.

**Table S2 Receiver operating characteristic parameter**

| No. | Lipid name | AUC | Ci1 | Ci2 | Specificity  (%) | Sensitivity  (%) | Threshold |
| --- | --- | --- | --- | --- | --- | --- | --- |
| 1 | PC(O-18:2/18:2) | 1 | 1 | 1 | 1 | 1 | 246822.04 |
| 2 | PC(O-18:2/18:1) | 1 | 1 | 1 | 1 | 1 | 243441.83 |
| 3 | PC(32:2) | 1 | 1 | 1 | 1 | 1 | 177837.08 |
| 4 | PC(O-18:2) | 1 | 1 | 1 | 1 | 1 | 343465.10 |
| 5 | PC(38:6) | 1 | 1 | 1 | 1 | 1 | 60111.42 |
| 6 | PE(38:3) | 1 | 1 | 1 | 1 | 1 | 527351.59 |
| 7 | LPE(O-18:2) | 1 | 1 | 1 | 1 | 1 | 114465.20 |
| 8 | PG(37:2) | 1 | 1 | 1 | 1 | 1 | 343.94 |
| 9 | PG(39:2) | 1 | 1 | 1 | 1 | 1 | 106775.26 |
| 10 | PI(38:4) | 1 | 1 | 1 | 1 | 1 | 87580.11 |
| 11 | SM(d36:1) | 1 | 1 | 1 | 1 | 1 | 190095.29 |

Area under the receiver operating characteristic (ROC) curve (AUC) was the area under ROC curve, Ci1 is the lower limit of AUC confidence interval; Ci2 is the upper limit of AUC confidence interval.

**Table S3 Data quality control of Illumina Paired-end sequencing and assembly as well as annotation on unigenes in different databases for Dezhou dongkey**

| Item | LDM | VAT |
| --- | --- | --- |
| Raw reads | 55625304±1731486 | 56904916±1201097 |
| Total raw bases (bp) | 8399420870±261454456 | 8592642249±181365688 |
| *After trimming (clean reads)* |  |  |
| Clean reads | 55118798±1721661 | 56402656±1200757 |
| Clean bases (bp) | 8105538257±256289176 | 8303496879±180659244 |
| Error rate(%) | 0.0239±0.0001 | 0.0241±0.0001 |
| Q20 (%) | 98.42±0.04 | 98.33±0.05 |
| Q30 (%) | 95.33±0.09 | 95.03±0.12 |
| GC content (%) | 54.33±0.11 | 50.70±0.14 |
| *After de novo assembly* |  |  |
| Total sequence base | 138936 | |
| Total unigenes num | 154948346 | |
| Average length | 1115.25 | |
| E90N50 | 6342 | |
| GC percent | 47.02 | |
| BUSCO score (%) | 82.30 | |
| Database | Annotation | Ration (%) |
| GO | 20587 | 14.82 |
| KEGG | 20300 | 15.61 |
| COG | 24000 | 17.27 |
| NR | 39202 | 28.22 |
| Swiss-Prot | 26119 | 18.80 |
| Pfam | 17537 | 12.62 |
| Total annotation | 40696 | 29.29 |
| Total | 138936 | 100 |

Q20 and Q30: the sequencing error rate 0.01 and 0.001 respectively.

GO, Gene Ontology; KEGG, Kyoto Encyclopedia of Genes and Genomes; COG, Clusters of Orthologous Groups; NR, Non-Redundant Protein Sequence Database.
